# Impact of Nitrate on the Removal of Pollutants from Water in Reducing Gas-Based Membrane Biofilm Reactors: A Review

**DOI:** 10.3390/membranes14050109

**Published:** 2024-05-09

**Authors:** Zhiheng Zhang, Zhian Huang, Haixiang Li, Dunqiu Wang, Yi Yao, Kun Dong

**Affiliations:** 1 College of Environmental Science and Engineering, Guilin University of Technology, 319 Yanshan Street, Guilin 541006, China; 19528364693@163.com (Z.Z.); huang2243416341@163.com (Z.H.); lihaixiang0627@163.com (H.L.); wangdunqiu@sohu.com (D.W.); 2 Guangxi Collaborative Innovation Center for Water Pollution Control and Safety in Karst Area, Guilin University of Technology, Guilin 541006, China; 3 Guangxi Key Laboratory of Theory and Technology for Environmental Pollution Control, Guilin 541006, China; 4 Guangxi Engineering Research Center of Comprehensive Treatment for Agricultural Non-Point Source Pollution, Guilin 541006, China; 5 Modern Industry College of Ecology and Environmental Protection, Guilin University of Technology, Guilin 541006, China

**Keywords:** microbial communities, denitrification, MBfR, composite pollutant

## Abstract

The membrane biofilm reactor (MBfR) is a novel wastewater treatment technology, garnering attention due to its high gas utilization rate and effective pollutant removal capability. This paper outlines the working mechanism, advantages, and disadvantages of MBfR, and the denitrification pathways, assessing the efficacy of MBfR in removing oxidized pollutants (sulfate (SO_4_^−^), perchlorate (ClO_4_^−^)), heavy metal ions (chromates (Cr(VI)), selenates (Se(VI))), and organic pollutants (tetracycline (TC), p-chloronitrobenzene (p-CNB)), and delves into the role of related microorganisms. Specifically, through the addition of nitrates (NO_3_^−^), this paper analyzes its impact on the removal efficiency of other pollutants and explores the changes in microbial communities. The results of the study show that NO^3−^ inhibits the removal of other pollutants (oxidizing pollutants, heavy metal ions and organic pollutants), etc., in the simultaneous removal of multiple pollutants by MBfR.

## 1. Introduction

Nitrate (NO_3_^−^) is the predominant pollutant found in Chinese water bodies, impacting 90% of shallow groundwater, with concentrations typically ranging from 10 to 100 mg/L. It is noteworthy that 70% of the Chinese population depends on this contaminated groundwater as their primary drinking water source [1,2]. In addition to NO_3_^−^, common pollutants include oxidizing pollutants (perchlorate (ClO_4_^−^), sulfate (SO_4_^−^)), heavy metal ions (chromate (Cr(VI)), selenate (SeO_4_^2−^)), and organic pollutants (tetracycline (TC), para-chloronitrobenzene (p-CNB)). Despite the low concentrations of these pollutants in water, they persist and pose a long-term threat to both the environment and human health [3,4,5].

The membrane biofilm reactor (MBfR) is an emerging water treatment technology that offers an effective solution to address water pollution problems. MBfR primarily utilizes hydrogen (H_2_) or methane (CH_4_) as electron donors to facilitate pollutant removal from water through microbial activity [6]. This technology offers not only high gas utilization efficiency and effective pollutant removal ability but also the advantages of simple operation and low energy consumption [7]. Currently, research on MBfR for the removal of individual pollutants is well-developed; however, in practical applications, it encounters complex combinations of multiple pollutants [8]. Denitrification, serving as a reduction pathway for NO_3_^−^, has undergone extensive research. Increasingly, researchers are focusing on the influence of NO_3_^−^ coexisting with other pollutants on the removal of these pollutants in MBfR [9,10]. The ongoing advancement of research on MBfR technology under complex combinations of pollutants is expected to yield significant advancements in the removal of micro pollutants, enhancement of sewage treatment efficiency, and overall improvement of water quality. Furthermore, the development and application of MBfR technology will provide crucial technical support for the realization of more sustainable and efficient sewage treatment systems.

## 2. The Basic Principles and Advantages and Disadvantages of MBfR

### 2.1. The Basic Principles of MBfR

The membrane biofilm reactor (MBfR) represents a novel approach to water treatment, integrating membrane separation technology with bioprocessing techniques. This method involves the arrangement of multiple hollow fiber membranes in a specific manner within a container to form a complete set of filtration and separation membrane components [11]. Hydrogen (H_2)_ or methane (CH_4_) serves as the electron donor to facilitate the reduction of oxidative pollutants in water. Depending on the application of the MBfR and the pore size of the hollow fiber membranes, these membranes can be categorized into microfiltration (MF), ultrafiltration (UF), nanofiltration (NF), and reverse osmosis (RO) membranes [12]. MF membranes are used to remove larger particles such as suspended solids, while UF membranes can eliminate certain viruses, bacteria, and some high-molecular-weight solutes. NF membranes, positioned between UF and RO, are capable of removing organic substances, some inorganic ions, and microbes. RO membranes, with the smallest pore sizes, can nearly remove all dissolved solids, organic materials, and microbes, making them suitable for the preparation of high-purity water, such as in desalination processes [13,14,15]. Depending on the membrane material, hollow fiber membranes can be divided into organic and inorganic membranes, as shown in Table 1. Given that the MBfR process primarily involves the separation of biomass and suspended solids in wastewater, the applications require high biocompatibility, effective filtration performance, and anti-fouling capabilities. Therefore, the hollow fiber membranes used in MBfRs are predominantly polymer organic membranes [16].

**Figure 1:**
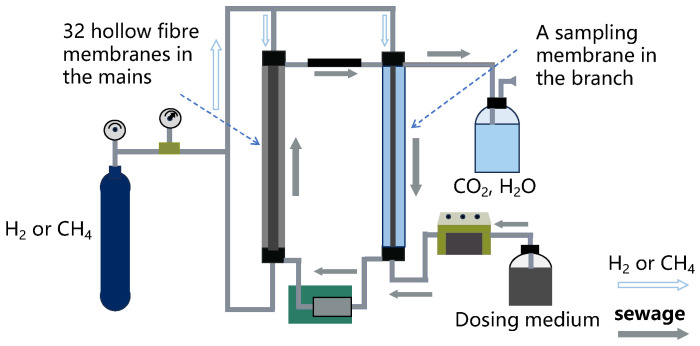
Schematic diagram of H_2_/CH_4_-MBfR.

The MBfR system, as depicted in Figure 1, directs H_2_ or CH_4_ from the top end of the reactor into the hollow fiber membranes that are secured within the tube to be utilized by microorganisms attached to the hollow fiber membranes in the left main pipe. Wastewater circulates through both columns, and as contaminants enter the main pipe, microorganisms are capable of effectively converting them into substances of lower toxicity or non-toxicity, concurrently producing harmless water and carbon dioxide. The branch on the right is tasked with separating the treated water and gases, facilitating the collection of samples for observation of the biofilm microorganisms. The water, subjected to microbial treatment, undergoes filtration through a hollow fiber membrane, while the generated gases are collected and discharged, enabling the recovery of valuable gases. This design not only ensures water quality compliance but also facilitates the valorization of gases [26,27,28].

### 2.2. Advantages of Membrane Biofilm Reactor (MBfR)

(1) Efficiency: The MBfR, through microbial metabolism and adsorption mechanisms, is capable of efficiently removing various types of pollutants, including oxidizing pollutants, heavy metal pollutants, and organic pollutants [29,30].
(2) Cost-effectiveness: Compared to traditional physical–chemical methods, MBfR has lower energy consumption and chemical usage. Additionally, by utilizing CH_4_ and H_2_ as electron donors, the demand for external carbon sources is further reduced, resulting in lower treatment costs [31].
(3) High gas utilization efficiency: Compared to the gas utilization efficiency of 5–50% in traditional wastewater treatment plants, MBfR achieves close to 100% utilization of electron donors (such as CH_4_ and H_2_) by utilizing a hydrophobic membrane and bubble-free aeration method [9,32].

### 2.3. Limitations of Membrane Biofilm Reactor (MBfR)

(1) High technical requirements: Due to the membrane biofilm reactor (MBfR) being an emerging technology involving multiple engineering and biological fields, strict control of parameters such as gas pressure, pH value, and biofilm thickness is necessary during operation to ensure higher removal efficiency [33].
(2) Stability issues of the biofilm: In the membrane biofilm reactor (MBfR), the biofilm is susceptible to environmental factors such as temperature, nutrient composition, and flow rate, which may result in membrane fouling, clogging, or failure, thereby affecting the treatment efficiency [7].
(3) Microbial balance risk: In the membrane biofilm reactor (MBfR), the stability of the microbial community is crucial for system performance. However, factors such as aeration pressure, reaction temperature, and changes in dissolved oxygen concentration in wastewater can lead to microbial imbalance. This imbalance may cause the overgrowth of dominant bacteria or the suppression of beneficial bacteria, thereby affecting the system’s stability and efficacy [34,35].

### 2.4. Comparison between H_2_-MBfR and CH_4_-MBfR

H_2_-MBfR and CH_4_-MBfR are both gas-permeable membrane-based bioreactor technologies. The difference lies in the utilization of different gases, H_2_ and CH_4_, as electron donors to facilitate the biodegradation of pollutants in wastewater. As a result, H_2_-MBfR and CH_4_-MBfR exhibit significant differences in terms of treatment efficiency, microbial community, and environmental impact. Regarding processing efficiency, comparing the NO_3_^−^ removal flux between CH_4_-MBfR and H_2_-MBfR, the results indicate that the NO_3_^−^ removal flux in CH_4_-MBfR is below 1.0 g N·m^−2^ d^−1^, while the NO_3_^−^ removal flux in H_2_-MBfR ranges from 1.1 to 3.9 g N·m^−2^ d^−1^; the data indicate that CH_4_-MBfR exhibits a nearly 4-fold lower NO_3_^−^ removal flux compared to H_2_-MBfR, as shown in Table 2. [36,37]. Under similar environmental pressures, introducing H_2_ as the electron donor in NO_3_^−^-polluted aquifers results in immediate and rapid absorption of H_2_. Denitrifying bacteria have an advantage in utilizing H_2_ for autotrophic growth [33,38,39], which accounts for the lower NO_3_^−^ removal flux in CH_4_-MBfR compared to H_2_-MBfR; Additionally, polydimethylsiloxane (PDMS) membranes have superior gas permeation characteristics compared to PP fibers [40,41]. The increased gas permeability allows the electron donor gas (such as H_2_) to transfer more effectively from the membrane to the biofilm where denitrification occurs, providing electrons for the denitrification pathway. Conversely, in the removal process of ClO_4_^−^, SO_4_^2−^, Cr(VI), and Se(VI), the removal flux of CH_4_-MBfR often exceeds that of H_2_-MBfR. Firstly, as seen in Table 2, during the removal process of ClO_4_^−^, SO_4_^2−^, Cr(VI), and Se(VI), the influent pollutant concentration or influent flux in CH_4_-MBfR is typically higher, directly resulting in higher removal flux. Secondly, as indicated in Table 3, methane oxidation generates higher energy, supporting robust microbial growth and contaminant reduction. Regarding microbial communities, in H_2_-MBfR, H_2_ is utilized as an electron donor to facilitate the growth of specific microbial communities, such as sulfate-reducing bacteria, methane-producing bacteria, and hydrogen-oxidizing bacteria [42,43]. In contrast, in CH_4_-MBfR, anaerobic methane-oxidizing bacteria, especially strains belonging to the Methylocystis genus, demonstrate significant growth advantages. Methanol-oxidizing denitrifying bacteria, such as Methloversatilis and Methylophilus, also play an important role in the denitrification process by utilizing intermediate organic compounds, such as methanol, produced during methane oxidation [44]. In terms of environmental benefits, H_2_-MBfR performs well in removing nutrients such as nitrogen and phosphorus from wastewater, thereby helping to prevent the occurrence of water eutrophication [44]. As the electron donor in MBfR, CH_4_ is generally more readily available and cost-effective compared to H_2_ [45,46]. CH_4_ is a byproduct of many natural processes and industrial activities, such as biogas production, while the production of H_2_ typically incurs higher costs and requires specialized manufacturing processes. Therefore, due to its low cost and wide availability, CH_4_ as an electron donor is attracting researchers’ attention in the field of biological reduction processes [47].

## 3. Pathways for Nitrate (NO_3_^−^) Reduction

In contemporary society, the use of agricultural fertilizers, animal excrement, and the discharge of various industrial and urban waste contribute to the continuous increase in nitrate (NO_3_^−^) concentrations in water bodies, becoming a widely prevalent environmental issue [55,56,57]. Especially in developing countries, the infiltration of NO_3_^−^ from septic tanks into groundwater has become a serious environmental challenge [58]. High environmental concentrations of nitrogen, such as NO^3−^, can cause eutrophication of water bodies. This eutrophication further promotes excessive growth of algae, leading to the occurrence of harmful algal blooms (HABs) and negatively impacting water quality and ecosystem health. In addition, high concentrations of NO_3_^−^ intake are associated with various health problems, including methemoglobinemia (blue baby syndrome), diabetes, and increased risk of infectious diseases [59]. To address these issue, various treatment methods for NO_3_^−^ in water bodies have been extensively studied, including physical methods such as ion exchange, reverse osmosis, and adsorption, chemical methods such as electrodialysis and electrochemical treatment, as well as biological methods such as microbial remediation and phytoremediation [60].

**Figure 2:**
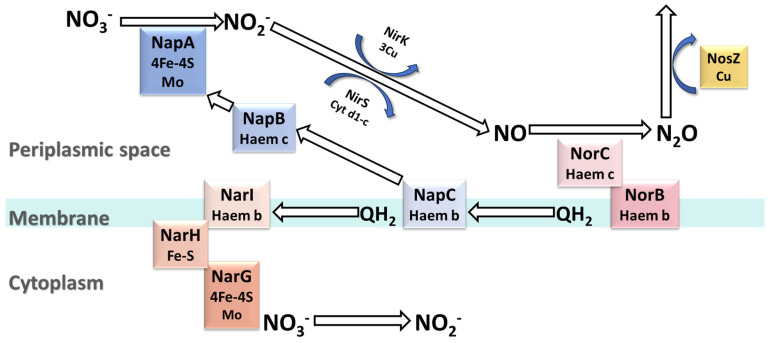
Nitrate reduction process. Abbreviations: Nar, membrane-bound nitrate reductase; Nap, periplasmic nitrate reductase; Nir, nitrite reductase; Nor, nitric oxide reductase; NosZ, nitrous oxide reductase; QH_2_, coenzyme Q, a quinone pool consisting of a storage of redox molecules related to coenzyme Q; Haem, heme; Fe-S, Fe-S clusters; cyt d1-c, a type of cytochrome complex. QH_2_, Haem, Fe-S, and cyt d1-c are primarily involved in electron transfer. Elements such as Mo and Cu mainly serve catalytic roles, working in conjunction with relevant reductases to complete reduction reactions.

In MBfR, the denitrification process involves a series of reduction reactions catalyzed by various enzymes, including Nar/Nap, NirS/NirK, NorB/NorC, and NosZ, and these enzymes catalyze a series of reduction reactions by accepting electrons supplied from H_2_ or CH_4_. Specifically, the H_2_-MBfR supports autotrophic denitrifying bacteria attached to hollow fiber membranes in utilizing H_2_ to reduce NO_3_^−^, while the CH_4_-MBfR requires methanotrophic bacteria within the biofilm to oxidize methane in order to drive the denitrification process [33]. The denitrification process is shown in Figure 2, in which the Nar and Nap enzymes play a role in reducing NO_3_^−^ to NO_2_^−^. Specifically, the membrane-bound NO_3_^−^ reductase Nar is a trimer composed of the following three subunits: NarG, NarH, and NarI. It receives electrons produced by autotrophic denitrifying bacteria or methanotrophic bacteria from the denitrifying bacteria’s quinone pool through NarI, then transfers them to the molybdenum cofactor (MoCo) active site of NarG via Fe-S clusters to achieve the reduction of NO₃⁻. Similarly, the periplasmic NO_3_^−^ reductase Nap transfers electrons to the activation site of NapA through NapC and NapB, achieving a similar function [8]. According to the research by Wang et al. [61], the Nar enzyme tends to respire under high concentrations of NO_3_^−^ compared to low levels, while the Nap enzyme exhibits the opposite behavior to achieve effective reduction of NO_3_^−^ at complementary concentrations. Furthermore, since the active site of Nar enzyme is located in the cytoplasm, NO_3_^−^ needs to be transported into the cytoplasm for denitrification through transport proteins. Under aerobic conditions, electrons may be captured by oxygen and participate in oxidation reactions, making the Nar enzyme more active in anaerobic environments. On the other hand, the activation site of Nap enzyme is located in the periplasm, making it more active in aerobic environments [62]. NirS and NirK are key enzymes that catalyze the reduction of NO_2_^−^ to the gaseous compound NO. Among them, NirK is a copper-containing NO_2_^−^ reductase, whereas NirS is a nitrite reductase containing cytochrome cd1. Despite their different catalytic mechanisms, NorB and NorC have the same function. NorB and NorC catalyze the reduction of NO to N_2_O, with NorB transferring electrons to the active site of NorC to facilitate the reaction [63]. Finally, the NosZ enzyme catalyzes the reduction of N_2_O to N_2_ [64,65].

## 4. The Impact of NO_3_^−^ on the Removal of Water Pollutants in MBfR

### 4.1. Oxidizing Pollutants

#### 4.1.1. Perchlorate Ions (ClO_4_^−^)

In recent years, there has been increasing attention paid to the potential impact of ClO_4_^−^ on human health due to their widespread use in the aerospace industry, illuminating flare, and firework manufacturing, among others [66]. The rising levels of ClO_4_^−^ in wastewater have raised concerns regarding its potential effects on human health. ClO_4_^−^ is recognized as one of the major pollutants in surface water and groundwater. It possesses significant oxidizing properties and has the potential to disrupt the endocrine system in the human body, particularly affecting thyroid function. Even at low concentrations, ClO_4_^−^ can potentially have adverse effects on human health [67,68]. Additionally, the coexisting pollutant commonly found with ClO_4_^−^ is NO_3_^−^. The impact of NO_3_^−^ presence on ClO_4_^−^ removal in MBfR has been extensively studied and discussed [69].

**Figure 3:**
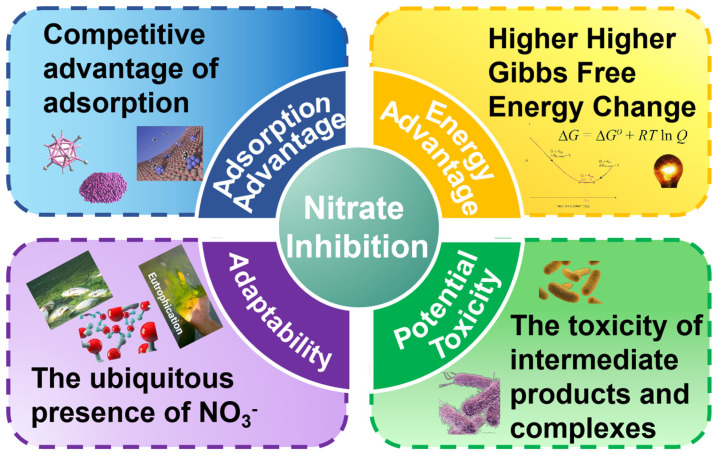
Analysis of the dominance of nitrate in the presence of other coexisting pollutants.

Lv et al. [69] discovered that in the CH_4_-MBBR system, when CH_4_ supply is abundant, the removal efficiency of 18 mg/L ClO_4_^−^ approaches 100%. After the addition of 15 mg/L NO_3_^−^, the reduction rate of ClO_4_^−^ decreased to 0.64 mmol/m^2^·d. However, after complete degradation of NO_3_^−^ at a rate of 2.76 mmol/m^2^·d, the reduction rate of ClO_4_^−^ increased to 1.68 mmol/m^2^·d, surpassing the rate observed in the presence of ClO_4_^−^ alone. Li et al. [70] investigated the impact of NO_3_^−^ reduction on ClO_4_^−^ removal in a H_2_/CO_2_-MBfR system under specific conditions. The reactor conditions included a H_2_ pressure of 0.04 MPa, CO_2_ pressure of 0.01 MPa, and a pH value of 7.2. They found that ClO_4_^−^ removal remained effective when the influent NO_3_^−^ concentration reached 10 mg/L. However, a further increase in NO_3_^−^ concentration, even with sufficient H_2_ supply, significantly decreased the efficiency of ClO_4_^−^ removal. It is worth noting that the removal rate of ClO_4_^−^ increased when the NO_3_^−^ concentration increased from 1 mg/L to 5 mg/L, possibly due to NO_3_^−^ acting as a nitrogen source that promotes microbial respiratory metabolism. Zhao et al. [71] reported that in a H_2_-MBfR system, when the electron donor H_2_ is limited, NO_3_^−^ competes with ClO_4_^−^ as an electron donor. In contrast, NO_3_^−^ inhibits the reduction of ClO_4_^−^ due to its adsorption advantage in the competition for adsorption sites and the adaptation caused by the prevalence of NO_3_^−^, as shown in Figure 3. According to these studies, an increase in NO_3_^−^ leads to the upregulation of denitrification genes narG, nirS, and ClO_4_^−^ reduction-related gene pcrA. However, when the electron donor is limited, a further increase in NO_3_^−^ does not induce changes in the related genes [69,71], indicating that the microbial growth in the system has reached its limit. Due to the lack of denitrification ability in the pcrA gene [72], an increase in NO_3_^−^ leads to a decrease in the ClO_4_^−^ reduction rate. However, after complete removal of NO_3_^−^, the increase in the ClO_4_^−^ removal rate may be attributed to the stimulation of rapid growth of perchlorate-reducing bacteria (PRB) by NO_3_^−^, resulting in a lag effect in ClO_4_^−^ reduction [73]. In a system where only ClO_4_^−^ is present, the presence of denitrification genes suggests that denitrifying bacteria can also participate in ClO_4_^−^ reduction [50]. A summary of the main enzymes, genes, and bacterial genera involved in the removal of various pollutants is provided in Table 4.

#### 4.1.2. Sulfate-Free (SO_4_^2−^)

In various industrial activities, such as mining, textile manufacturing, dye production, and flue gas desulfurization, the generation of wastewater containing SO_4_^2−^ ions is a common occurrence [79]. In drinking water, excessive levels of SO_4_^2−^ content can potentially lead to health issues such as allergic reactions and diarrhea [87,88]. Furthermore, this type of industrial wastewater often contains various metal pollutants. The biological process employed for treating this wastewater primarily involves the transformation of SO_4_^2−^ into hydrogen sulfide (H_2_S). This process not only aids in the precipitation of certain metals but also facilitates the further oxidation of H_2_S into elemental sulfur (S_0_), effectively removing SO_4_^2−^ from the wastewater [51].

According to the study conducted by Alex Schwarz et al. [79], they found that in a H_2_-MBfR system with a surface loading of 2.1 g/m^2^-d, the removal efficiency of SO_4_^2−^ was close to 100%. However, when the influent load is doubled, the removal efficiency significantly decreases due to the limitation of H_2_ concentration. Upon increasing the H_2_ pressure from 2 psig to 10 psig, the removal efficiency is restored. Zhou et al. [89] successfully achieved simultaneous removal of nitrate (NO_3_^−^), sulfate (SO_4_^2−^), and selenate (Se(VI)) in the H_2_-MBfR system. They found that while almost complete removal was achieved for NO_3_^−^ and Se(VI) at load rates of 10 mg/L and 2 mg/L, respectively, the effluent concentration of SO_4_^2−^ was close to 50 mg/L at a load rate of 50 mg/L. Upon extending the hydraulic retention time (HRT) from 5.2 h to 10.4 h, the electron consumption rate of SO_4_^2−^ increased from 1.4 mmol e/day to 4.7 mmol e/day. It is understandable that with a longer reaction time, a higher removal rate of SO_4_^2−^ can be achieved. Compared to NO_3_^−^ and Se(VI), the lower removal rate of SO_4_^2−^ is mainly due to its lower Gibbs free energy, which thermodynamically hinders the forward progress of the reaction. Table 3 shows the Gibbs free energy changes of various pollutant reactions and Figure 3 shows the energetic advantages of NO_3_^−^. During the initial stage of the H_2_-MBfR reaction, Aura Ontiveros-Valencia [90] and colleagues observed that when the concentrations of NO_3_^−^ and SO_4_^2−^ were 10 mg/L and 46 mg/L, respectively (Stage 1), the removal rate of NO_3_^−^ was close to 100%, while the effluent concentration of SO_4_^2−^ was close to 46 mg/L. NO_3_^−^ exhibits a significant inhibitory effect on the reduction of SO_4_^2−^. As the influent NO_3_^−^ concentration changed to 20 mg/L (Stage 2), the population of denitrifying bacteria (DB) increased, while the sulfate-reducing bacteria (SRB) slightly decreased. When the NO_3_^−^ concentration was reduced to 5 mg/L (Stage 3) and 1 mg/L (Stage 4), compared to Stage 1, the quantity of DB did not decrease, but the NO_3_^−^ concentration was lower. The higher ratio of denitrification genes to NO_3_^−^ concentration indicated the upregulation of denitrification genes, leading to the rapid reduction of NO_3_^−^ and the alleviation of the inhibitory effect on SRB [91]. Therefore, it can be observed that the removal rate of SO_4_^2−^ in Stage 3 is greater than 75%, while in Stage 4, it is greater than 90%.

### 4.2. Heavy Metal Ions

#### 4.2.1. Chromate (Cr(VI))

Chromium (VI) in water poses significant risks to human health due to its carcinogenic, mutagenic, and teratogenic properties. Specifically, the excessive presence of Cr(VI) in drinking water can severely impact liver function, kidney function, and cognitive function [92,93]. Certain specific microorganisms are capable of biologically reducing Cr(VI) to Cr(III), which subsequently precipitates as Cr(OH)_3_, enabling effective removal. Similarly, denitrifying bacteria can also reduce NO_3_^−^ to N_2_(g). Compared to traditional physical and chemical remediation methods, the bioreduction processes of Cr(VI) and NO_3_^−^ offer economic and environmental advantages, attracting widespread attention [94].

Chung et al. [95] found that in the H_2_-MBfR system, as the influent NO_3_^−^ concentration increased from 0 mg/L to 10 mg/L, the reduction rate of Cr(VI) decreased from 80% to 40%, while the effluent NO_3_^−^ concentration remained relatively stable. Under conditions of 29 ± 1 °C, pH of 7.0–7.5, and sufficient CH_4_ supply, Zhong et al. [96] investigated the impact of NO_3_^−^ on the removal of Cr(VI) by CH_4_-MBfR. Research has shown that in the CH_4_-MBfR system with Cr(VI) as the sole electron acceptor and a removal rate of 100% (Stage 1), the introduction of 2.2 mg/L of NO_3_^−^ leads to a significant decrease in the quantity of Meiothermus and a reduction in Cr(VI) removal rate to less than 25% (Stage 2). However, after removing the surface load of NO_3_^−^, the removal rate of Cr(VI) recovers to 70% (Stage 3), and the quantity of Meiothermus also shows some recovery. Subsequently, when reintroducing 0.7 mg/L of NO_3_^−^ (Stage 4), Meiothermus sharply decreases again, and the removal rate of Cr(VI) drops to approximately 60%. By increasing the liquid circulation rate in the reactor 1.5 times to reconstruct the biofilm, the reduction rate of Cr(VI) stabilizes at 80% (Stage 6). Although there are currently no reports on the interaction between NO_3_^−^ and Meiothermus, the data suggest that NO_3_^−^ may have a significant inhibitory effect on the growth of Meiothermus. Compared to the dominant role of Meiothermus in Stage 1, Pelomonas gradually enriches after the addition of NO_3_^−^ and reaches its peak in Stage 6, indicating the potential of Pelomonas as a Cr(VI) reducer after the addition of NO_3_^−^ [96]. The reduction of Cr(VI) is the result of synergistic interactions among various microorganisms. In the CH_4_-MBfR system, CH_4_ generates intermediate products such as methanol, lactic acid, and acetic acid under the action of methane-oxidizing bacteria. Chromium-reducing bacteria like Meiothermus utilize these intermediate products to reduce Cr(VI) [31]. The reduction products of Cr(VI) mainly form extracellular polymeric substances (EPS) or Cr(OH)_3_. Additionally, a small amount of Cr(III) ions combine with negatively charged groups inside Meiothermus cells, forming intracellular precipitates. Studies have shown that the introduction of NO_3_^−^ can affect the community structure of Cr(VI)-reducing bacteria, thereby altering the rate of Cr(VI) reduction [97].

#### 4.2.2. Selenate (Se(VI))

Selenium is a trace element that is crucial to human health, and its intake needs to be balanced between maintaining health and environmental exposure [98]. Selenate (Se(VI)) and selenite (Se(IV)) are the primary soluble forms of selenium, and their biological reduction pathway typically involves the conversion from Se(VI) to Se(IV) and further to Seo [99]. In recent years, the increasing industrial activities have led to a continuous rise in selenium levels in water environments, which has raised significant concerns due to its substantial potential for bioaccumulation. The high toxicity of soluble forms of selenium in water to organisms has prompted extensive research on the removal of Se(VI) in MBfR [100,101].

In the study conducted by Xia et al. [99], the initial removal efficiency of Se(VI) in a H_2_-MBfR influent system containing 10 mg/L of NO_3_^−^ and 2 mg/L of Se(VI) was approximately 75%. After 20 days, it increased to around 95%. The anaerobic biofilm community in this system was capable of simultaneous removal of Se(VI) and NO_3_^−^. In the study conducted by Lai et al. [102], it was found that after the addition of 10 mg/L of NO_3_^−^ and 1 mg/L of Se(VI) (Stage 2), there was a restructuring of the microbial community compared to the situation without the addition of NO_3_^−^ (Stage 1) and it was found that the proportion of β-Proteobacteria increased from an initial 55% to 90%, while the proportion of Methyloversatilis decreased. In Stage 3, where the influent contained only 1 mg/L of Se(VI) without NO_3_^−^, the quantity of Methyloversatilis increased several-fold, indicating its preference for respiratory Se(VI) over NO_3_^−^. Due to limitations in electron donor flux, the removal efficiency of Se(VI) decreased to less than 10% in Stage 2, where NO_3_^−^ was present. However, in Stage 3 without NO_3_^−^, the removal efficiency of Se(VI) recovered to 60%. As mentioned earlier, *Dechloromonas* can utilize ClO_4_^−^ for metabolism, and during the reduction process of Se(VI), *Dechloromonas* also produces Se(VI) reductase. In the CH_4_-MBfR system, Lai et al. [103] discovered that 70% of Se(VI) could be reduced to Se_0_. In contrast, the conversion rate using H_2_ as the electron donor was only 40%. When the influent contained 10 mg/L of NO_3_^−^ and 1 mg/L of Se(VI), due to limited CH_4_ flux, 50% of Se(VI) was converted to Se(IV), and 10% was converted to SeO, resulting in a total removal of 60% of Se(VI), while the removal efficiency of NO_3_^−^ was 70%. In comparison to the other pollutants mentioned earlier, the removal efficiency of NO_3_^−^ remained higher when the electron donor was limited, and the removal efficiencies of Se(VI) and NO_3_^−^ decreased in parallel. Comamonadaceae is an important bacterial genus in the process of Se(VI) reduction. Compared to SRB (sulfate-reducing bacteria), the abundance of Comamonadaceae decreased synchronously with an increase in NO_3_^−^ concentration, while SRB remained relatively stable due to their metabolic diversity [90].

### 4.3. Organic Matter

#### 4.3.1. Tetracycline (TC)

TC is a widely used antibiotic in humans and animals, and reports have shown that tetracycline can enter the environment through human and animal urine and feces. This emission process resulted in tetracycline pollution in the environment. The presence of tetracycline may pose a potential threat to aquatic or soil ecosystems. For instance, high concentrations of tetracycline exhibit toxicity to algae and zooplankton in aquatic environments, which may disrupt their growth and reproduction processes [104,105]. In soil environments, tetracycline affects the structure and function of soil microbial communities through processes such as adsorption, degradation, and migration. Currently, the removal efficiency of tetracycline in wastewater treatment plants is not satisfactory. However, research on the MBfR technology has shown promising progress [106,107].

Salman et al. [108] researchers compared the efficiency of H_2_-MBfR and O_2_-MBfR in removing TC by adjusting the HRT. The researchers found that when the HRT was decreased from 10 h to 1 h, TC removal decreased from 92% to about 16% or so in H_2_-MBfR, whereas in O_2_-MBfR, removal decreased from 52% to 0%. In addition, the researchers found that denitrification and nitrification rates were stable at 85% to 99% in all cases except for HRT of 1 h. This indicates that the microbial community responsible for denitrification and nitrification in the MBfR system remained relatively resilient and was able to maintain its metabolic activities under different HRT conditions. Taşkan et al. [109] found that TC removal was around 63% at an HRT of 18 h and O_2_ pressure of 0.41 bar. Reducing both HRT and aeration pressure resulted in a decrease in TC removal, while nitrification remained relatively intact. In another study of H_2_-MBfR, a similar pattern was found by Taşkan et al. [5]. H_2_-MBfR, as a system with H_2_ as an electron donor, was superior in tetracycline removal than TC removal with O_2_ as an electron acceptor. And the longer the contact time of microbial biofilm with TC, the higher the removal rate. On the other hand, lowering the HRT in the O_2_-MBfR system resulted in a drastic decrease in TC removal from 52% to 0. These findings emphasize the importance of optimizing the HRT in the MBfR system for maximum TC removal. Pseudomonas was mentioned above as a Cr(VI)-reducing bacterium. Certain strains of the genus Pseudomonas also exhibit resistance and metabolism to TC. They are able to utilize TC as a carbon source for growth and efficiently remove TC from water. Similar to the removal of oxidizing and metal pollutants, NO_3_^−^ also exhibits the stronger competition shown in Figure 3 in the removal of tetracycline. The major transformation products of TC are ETC, EATC and ATC [109].

#### 4.3.2. p-Chloronitrobenzene (p-CNB)

A significant risk to the environment due to its persistence and high toxicity is p-CNB. It is also associated with methemoglobinemia in humans [110]. p-CNB is present in industrial wastewater in a highly stable and low biodegradable form. It has significant carcinogenic and mutagenic properties [111,112]. In the H_2_-MBfR system, p-CNB can be bioreduced to p-CAN and further dechlorinated to produce aniline (AN). The low environmental and human health hazards of these AN compounds make the H_2_-MBfR technology a promising approach for treating p-CNB pollution in industrial wastewater.

Li et al. [113] discovered that under the conditions of an influent NO_3_^−^ concentration of 5 mg/L and a p-CNB concentration of 1000 μg/L, the effluent p-CNB concentration from the H_2_-MBfR system was approximately 60 μg/L, achieving a removal rate of 94%. Furthermore, with increasing NO_3_^−^ concentration, the concentrations of p-CNB and p-CAN in the effluent increased while the AN concentration decreased. When the influent NO_3_^−^ concentration was 50 mg/L, the effluent p-CNB concentration was 130 μg/L, with a removal rate of 87%, while the NO_3_^−^ removal rate was only 60%. According to Table 3, the conversion of p-CNB to p-CAN exhibits a higher change in free energy, which thermodynamically explains why the removal rate of p-CNB is higher than denitrification. Xia et al. [114] observed that in a H_2_-MBfR system with a H_2_ pressure of 0.04 MPa and an HRT of 4.8 h, the removal rate of NO_3_^−^ exceeded 90%. However, when 2 mg/L of p-CNB was added, the removal rate of NO_3_^−^ gradually decreased to 70%, while the removal rate of p-CNB reached 85%. With the addition of 2 mg/L of p-CNB, the removal of NO_3_^−^ gradually decreased to 70%, while the removal of p-CNB reached 85%. During the reduction of p-CAN to AN, NO_2_^−^, NO and N_2_O produced by denitrification may react with heme and non-heme iron-containing proteins to form harmful substances [115,116] such as heme-NO_x_. These toxic substances may inhibit the reductive dechlorination of p-CAN and this is the potential toxicity shown in Figure 3. In addition, N_2_O accumulation due to the fact that many microorganisms do not possess N_2_O reductase [8] is one of the reasons [117] for the inhibition of p-CAN reductive dechlorination. Hydrogenophilic bacteria play a key role in p-CNB removal, as these bacteria first adsorb p-CNB on their surface and then use H_2_ as an electron donor to convert p-CNB to the safer compound p-CAN through a series of biochemical reactions. During this process, the bacteria are able to generate ATP, which allows them to obtain energy to support their growth and reproduction.

In summary, the inhibitory effect of NO3- on the removal of other pollutants is illustrated in Figure 3.

## 5. Conclusions and Outlook

This review article provides insight into the effectiveness of MBfR in eliminating oxidizing pollutants, heavy metal ions, and organic contaminants. Although MBfR technology is quite mature in treating single pollutants, it faces challenges when multiple pollutants coexist. Specifically, NO_3_^−^ tends to inhibit the removal of other pollutants for several reasons, as outlined below:

(1) NO_3_^−^ is more advantageous in competing with other pollutants for the same adsorption sites, thus reducing the removal efficiency of other pollutants.
(2) Reactions involving NO_3_^−^ typically have higher Gibbs free energies, making them more attractive for microbial metabolism.
(3) Given the prevalence of nitrate, many microbial communities may have adapted to use NO_3_^−^ as their primary electron acceptor due to its higher affinity coefficient.
(4) Denitrification intermediates such as NO_2_^−^, NO, N_2_O, and their complexes formed with metal ions or proteins may poison microorganisms, affecting the efficiency of MBfR in removing pollutants.

For future MBfR research, the focus could shift to understanding the interactions between different pollutants, constructing multi-pollutant systems, and elucidating synergistic and antagonistic mechanisms. Studying changes in microbial communities when treating multiple pollutants, identifying potential mechanisms of action, and analyzing microorganisms capable of producing a range of catalytic enzymes could all contribute to improving wastewater treatment efficiency. Although research on MBfR treatment of oxidizing and metal pollutants is relatively mature, the mechanisms of organic pollutant removal by MBfR still require in-depth study. Upcoming studies could examine the principles of organic pollutant removal, how various chemical bonds are broken, removal of organic and inorganic pollutants under coexistence, and the role of relevant microbial communities in the removal of organic pollutants.

## Figures and Tables

**Table 1:** Characteristics of common organic and inorganic hollow fiber membranes.

The Membrane Materials		Feature	Reference
Organic membranes	Polysulfone (PSF)	It exhibits excellent chemical stability and mechanical strength, rendering it applicable for diverse water treatment applications.	[17]
	Polyethersulfone (FES)	It possesses outstanding heat resistance and chemical resistance, along with good film-forming performance and cost-effectiveness.	[18,19]
	Polyvinylidene fluoride (PVDF)	It exhibits excellent chemical and physical durability, as well as biocompatibility.	[20,21]
	Polyaniline (PANI)	It exhibits high electrical conductivity and is frequently utilized as a component in blends/composites or as a coating on polymer films.	[22]
	Polypropylene (PP)	It is a lightweight, low-cost thermoplastic polymer that exhibits good chemical stability and mechanical performance.	[23]
Inorganic membranes	Ceramic	It is known for its high-temperature stability, excellent chemical stability, and long service life, making it suitable for filtration in high-temperature processes and aggressive corrosive environments.	[24]
	Metal	Due to its exceptional mechanical strength and temperature resistance, it is frequently employed in gas separation and certain specific chemical processes.	[25]

**Table 2:** Comparison of H_2_-MBfR and CH_4_-MBfR in terms of removal flux.

		Hollow Fiber Membrane Types	Shared Pollutants	Environmental Pressure (MPa)	pH	T (°C)	HRT (h)	Influent Concentration (mg/L)	Influent Flux (g·m^−2^ d^−1^)	Removal Flux (g·m^−2^ d^−1^)	References
NO_3_^−^	H_2_-MBfR	Non-porous PDMS fibers	None	0.104	7–8	22	4	-	13.6	3.300	[37]
	CH_4_-MBfR	Non-porous PP fiber	None	0.114	-	-	12	25	-	0.460	[48]
ClO_4_^−^	H_2_-MBfR	Non-porous PP fiber	3 mg/L NO_3_^−^ 30 mg/L SO_4_^2−^	0.223	7.4–7.8	-	-	0.09	0.0065	0.0065	[49]
	CH_4_-MBfR	Microporous polyethylene fiber	None	0.020	7.2–7.6	31 ± 1	24	-	0.1068	0.093	[50]
SO_4_^2−^	H_2_-MBfR	Non-porous PP fibers	None	0.138	8–8.86	21 ± 3	-	-	1.9	0.830	[51]
	CH_4_-MBfR	Mitsubishi Rayon (model MHF-200TL, Mitsubishi Rayon Co., Ltd., Tokyo, Japan)	1 mg/L Cr(VI)	0.069	7.0–7.5	29 ± 1	-	1	2.55	1.004	[52]
Cr(VI)	H_2_-MBfR	Mitsubishi Rayon (Model MHF 200TL, Mitsubishi Rayon Co., Ltd., Tokyo, Japan)	5 mg/L NO_3_^−^ 80 mg/L SO_4_^2−^	0.017	-	-	-	0.25	-	0.034	[53]
	CH_4_-MBfR	Mitsubishi Rayon (Model MHF 200TL, Mitsubishi Rayon Co., Ltd., Tokyo, Japan)	None	0.069	6.8–7.5	35 ± 1	-	2	-	0.070	[46]
Se(VI)	H_2_-MBfR	Mitsubishi Rayon (Model MHF 200TL, Mitsubishi Rayon Co., Ltd., Tokyo, Japan)	5 mg/L NO_3_^−^ 80 mg/L SO_4_^2−^	0.017	-	-	-	0.25	-	0.031	[53]
	CH_4_-MBfR	Microporous polyethylene fiber	None	0.069	7.0–7.4	35 ± 1	2.7	5	0.529	0.182	[54]

**Table 3:** Gibbs free energy of reduction reaction for removal of common pollutants.

Pollutants	Chemical Reaction	ΔGo’ (kJ e^−1^)
Nitrate (NO_3_^−^)	2NO_3_^−^ + 6H_2_ → N_2_ + 6H_2_O	−112
Nitrate (NO_3_^−^)	8NO_3_^−^ + 5CH_4_ + 8H^+^ → 5CO_2_ + 4N_2_ + 14H_2_O	−765
Perchlorate (ClO_4_^−^)	ClO_4_^−^ + 4H_2_ → Cl^−^ + 4H_2_O	−118
Perchlorate (ClO_4_^−^)	ClO_4_^−^ + CH_4_ → Cl^−^ + 2H_2_O + CO_2_	−941
Sulfate (SO_4_^2−^)	SO_4_^2−^ + 5H_2_ → H_2_S + 4H_2_O	−19
Chromate (Cr(VI))	CrO_4_^2−^ + 1.5H_2_ + 2H^+^ → Cr(OH)_3_ + H_2_O	−9
Chromate (Cr(VI))	8CrO_4_^2−^ + 3CH_4_ + 16H^+^ → 3CO_2_ + 4Cr_2_O_3_ + 14H_2_O	−708
Celenate (SeO_4_^2−^)	SeO_4_^2−^ + CH_4_ → Se_0_ + 2H_2_O	−71
Tetracycline (TC)	C_22_H_24_N_2_O_8_ + 43H_2_ → 22CH_4_ + 2NH_3_ + 8H_2_O	/
p-chloronitrobenzene (p-CNB)	p-CNB +2H_2_ → p-CAN + 2H_2_O	−122.7

**Table 4:** Enzymes related to the removal of common pollutants.

Functional Enzymes	Gene	Genus	References
Nitrate reductase	NapA, NarG	*Thauera*	[74,75]
Nitrite reductase	NirK, NirS	*Thauera*, *Mesorhizobium*, *Cycloclastes*	[75,76]
Nitric oxide reductase	NorB, NorC	*Ps. Stutzeri*, *Paracoccus denitrificans*	[75]
Nitrous oxide reductase	NosZ	*Paracoccus pantotrophus*	[75,77]
Perchlorate reductase	PcrA	*Dechloromonas*	[78]
Sulfate reductase	DsrA	*Desulfovibrio*, *Desulfomicrobium*	[79,80]
Chromate reductase	ChrR	*Pseudomonas putida*	[81]
Selenite reductase	SerA	*T. Selenatis*, *Pseudoxanthomonas*	[82,83]
Tetracycline-degrading enzyme	Tet(X)	*Pichia pastoris*	[84]
Nitroreductase	Psntr	*Psychrobacter* sp.	[85]
Dehalogenase (enzyme)	PcbA4, PcbA5	*Dehalococcoides*, *Dehalobacter*	[86]

## Data Availability

Not applicable.
